# Motivational Mechanisms and Outcome Expectancies Underlying the Approach Bias toward Addictive Substances

**DOI:** 10.3389/fpsyg.2012.00440

**Published:** 2012-10-22

**Authors:** P. Watson, S. de Wit, Bernhard Hommel, R. W. Wiers

**Affiliations:** ^1^ADAPT Lab, Department of Developmental Psychology, University of AmsterdamAmsterdam, Netherlands; ^2^Cognitive Science Center Amsterdam, University of AmsterdamAmsterdam, Netherlands; ^3^Department of Clinical Psychology, University of AmsterdamAmsterdam, Netherlands; ^4^Cognitive Psychology Unit, Leiden UniversityLeiden, Netherlands; ^5^Leiden Institute for Brain and CognitionLeiden, Netherlands

**Keywords:** approach, dual-process theory, addiction, associative learning, motivation, goal-directed action, habit, Pavlovian-instrumental transfer

## Abstract

Human behavior can be paradoxical, in that actions can be initiated that are seemingly incongruent with an individual’s explicit desires. This is most commonly observed in drug addiction, where maladaptive behavior (i.e., drug seeking) appears to be compulsive, continuing at great personal cost. Approach biases toward addictive substances have been correlated with actual drug-use in a number of studies, suggesting that this measure can, in some cases, index everyday maladaptive tendencies. At present it is unclear whether this bias to drug cues is a Pavlovian conditioned approach response, a habitual response, the result of a Pavlovian-instrumental transfer process, or a goal-directed action in the sense that expectancy of the rewarding effects of drugs controls approach. We consider this question by combining the theoretical framework of associative learning with the available evidence from approach bias research. Although research investigating the relative contributions of these mechanisms to the approach bias is to date relatively limited, we review existing studies and also outline avenues for future research.

## Introduction

To what extent is human behavior under voluntary control? Drug addiction is an extreme example, where drug seeking continues despite negative social or interpersonal consequences. Although many drug users are fully aware of the negative consequences and seek treatment in order to abstain from drug use, risk of relapse remains high. This highlights the paradoxical, destructive characteristic of addiction: that drug-seeking behavior persists despite explicit motivations to the contrary. Understanding the cognitive and motivational mechanisms that maintain such behaviors may allow us to better understand action control in general.

The approach bias is a behavioral inclination to approach rather than avoid certain stimuli. Experimental research into the approach bias has provided evidence for correlations with actual drug use and it is theorized that an approach bias may contribute to problematic drug-related behavior (Stacy and Wiers, 2010). An important question that remains to be addressed, however, is what the approach bias represents and how it relates to other features of drug use such as craving. Specifically, it is not clear whether the approach bias has the characteristics of being a goal-directed behavior, controlled by the expectancy of a rewarding outcome. Alternatively, it may better fit the profile of a Pavlovian conditioned response, or of a persistent, habitual response to drug cues, or it may be driven by Pavlovian-instrumental interactions.

In the present paper, we provide a comprehensive overview of these possible mechanisms that may facilitate the approach bias toward addictive substances. To this end, we relate the approach bias to theories of addiction as well as theoretical concepts of associative learning theory (based on fundamental animal as well as human behavioral research). Furthermore, we critically review the experimental measurement of approach bias and the evidence that it can underlie maladaptive behaviors. The existing literature does not allow firm conclusions to be drawn about the relative contributions of different mechanisms, although we hope that this manuscript will inspire empirical investigations of this issue. To further stimulate such investigations, we will outline several possible avenues for future research at the end of this article.

## Theoretical Background

Addiction has been described as a progressive neurological disorder of learning and memory whereby everyday associative learning processes become pathological (Hyman, 2005; Koob and Volkow, 2010). Teenagers experimenting with alcohol, for example, may discover that it makes them feel sociable and lively. The initial learning of associations between rewarding outcomes and the contexts or behaviors that lead to them, allows for the emergence of goal-directed behavior (e.g., approaching the bar at a party *in order* to feel sociable). For many teenagers, this goal-directed behavior can over time become habitual, such that simply being in a party context is the impetus to move toward the bar, regardless of any consideration of possible (pleasant) outcomes. For some individuals it may ultimately also persist when this behavior has undesirable consequences. Those individuals that continue to consume alcohol despite pervasive negative consequences and sometimes even explicit intentions to abstain may be regarded as *compulsive* drug-users (Koob and Volkow, 2010).

A number of learning and reward processes underlie this behavioral transition from voluntary drug use to clinically recognized drug dependency whereby the consumption is maintained at increasingly high cost. Associative learning theories, arising from the systematic study of animal behavior and neurobiology, offer a coherent framework for defining and dissociating these processes (see Balleine et al., 2008; Balleine and O’Doherty, 2009). Initial empirical investigations in humans support the case for applying the associative framework to human behavior (review: de Wit and Dickinson, 2009). The psychological constructs arising from associative learning theory are also paralleled in some neurocomputational models of decision-making, and we refer the interested reader to recent publications on this topic for an in-depth discussion (Daw et al., 2005; Dayan et al., 2006; Balleine et al., 2008). We shall discuss approach behavior within the associative learning context, because this context provides the most useful concepts to understand and investigate approach and avoidance behavior. At the same time, however, we emphasize that a purely non-propositional, associative framework can be argued to be insufficient as an exhaustive account of human decision-making (see for example Mitchell et al., 2009, but see also associated commentaries).

### Pavlovian or classical conditioning

Associations between environmental cues (e.g., beer at a party) and motivationally relevant events (e.g., feeling lively and sociable) are acquired over time and may play an important role in guiding everyday decision-making. The conditions under which such associations emerge have been studied by means of Pavlovian (or classical) conditioning methods that establish a predictive relationship between these (Pavlov, 1927). Conditioned stimuli (CS) are cues that were once irrelevant (e.g., a food bowl) and that through repetitive pairing with a motivationally relevant stimulus (e.g., food) come to elicit conditioned responses. These conditioned responses can be consummatory in nature (e.g., salivation) or preparatory (approach toward the food bowl; Konorski, 1967). Furthermore, it has been shown in humans that Pavlovian conditioning can lead to acquired likes and dislikes of previously neutral objects and places (evaluative conditioning; Hermans et al., 2002; Hofmann et al., 2010). These processes may play an important role in drug-seeking behavior as contexts that were previously paired with the rewarding experience of drug taking become preferred and will elicit conditioned approach responses that may support drug seeking.

### Pavlovian versus instrumental learning

While conditioned approach may well contribute to the approach bias, there may also be an instrumental component. Whereas Pavlovian behavior results from the contingencies between stimuli and motivationally relevant events, instrumental behavior arises from the contingency between a response and a motivationally relevant outcome (Skinner, 1938). If the outcome is rewarding, the instrumental agent will acquire the responses that lead to this outcome.

Embedded within any instrumental contingency is also a Pavlovian relationship between the context and the outcome. For example, where a light stimulus (S) may signal that a lever press response (R) will produce a food pellet outcome (O) there is an inherent, parallel S–O relationship between the light and the rewarding food pellet being conditioned. Often a conditioned Pavlovian response will facilitate instrumental behavior (e.g., salivation or approach to the food bowl will facilitate eating behavior). There are occasions, however, when contradicting instrumental and Pavlovian responses (whether preparatory or consummatory) can cause conflict (Sheffield, 1965; Hershberger, 1986). Hershberger (1986), for example, created a “looking glass world” in which a food bowl receded with twice the speed at which hungry chicks ran toward it, and drew near at twice the speed at which the chicks ran away from it. To gain access to the food the chicks had to learn to overcome the Pavlovian bias to approach the food bowl, which acted as a CS for the food it contained. Most chicks continued to run toward the bowl, however, and thereby lost the available food. The approach bias of these chicks was clearly controlled predominantly by Pavlovian conditioning, as sensitivity to the R–O contingency should have allowed them to learn to make the opposite response of running away from the food bowl. On the other hand, it is well-known that animals, as well as humans, are capable of instrumental behavior. In a later section we will review the evidence for an instrumental component of the approach bias in humans as measured in the laboratory.

### From goal-directed actions to habitual responses

Instrumental conditioning could contribute to the approach bias by giving rise to either goal-directed approach or to habitual approach that is triggered directly by environmental stimuli. Goal-directed actions are performed in order to achieve desirable outcomes (e.g., approaching the bar at a party to feel more lively and sociable) and are thus flexibly modulated by the incentive value of the outcome (Adams and Dickinson, 1981). Over time, however, these appetitive outcomes gradually reinforce S–R associations, that give rise to habitual responding that is directly evoked by the context. In this scenario, the party context triggers approach, rather than consideration of the drinking outcome. Overtraining of an instrumental action is one way to bring about habitual responding (Adams, 1982; Dickinson, 1985; Tricomi et al., 2009). In the early stages of drug use, drug-seeking behavior appears to meet the criteria for goal-direction action. Habitual drug seeking triggered by certain cues and contexts may, however, help to maintain drug-seeking behavior, even when the drug is no longer desired.

In animal studies, the degree to which behavior is goal-directed or habitual is formally assessed by means of the outcome devaluation procedure. In this procedure instrumental training is followed by devaluation of the instrumental outcome (e.g., through satiation on a particular food reward). Subsequently, an extinction test is conducted to assess instrumental responding for the devalued outcome. If behavior is predominantly under goal-directed control, responding for the devalued outcome should be immediately reduced. In contrast to goal-directed behavior, S–R habits are not sensitive to devaluation of outcomes and such behavior will persist.

### Pavlovian-to-instrumental transfer

A popular beer brand logo can prompt thoughts of beer drinking, which may increase the probability that an individual will head to the nearest bar and realize that outcome. This anticipatory effect is formally described as Pavlovian-to-instrumental transfer (PIT). In lab demonstrations of this phenomenon, a common outcome (such as a food reward) functions as both a Pavlovian reward and also an instrumental reward, in separate training phases. This training allows for the separate development of both Pavlovian S–O expectancies and instrumental O–R associations. The interaction effect is then assessed by presenting the Pavlovian cue whilst the subject is given the opportunity to perform the instrumental response for that outcome. Many studies have shown that although the Pavlovian cue was never directly paired with a response, the expectancy elicited by the cue increases the likelihood of instrumental responding for that specific reward (outcome-specific PIT) or in some cases boosts responding generally (general PIT; Estes, 1948; Rescorla and Solomon, 1967; Corbit and Balleine, 2005). PIT effects are now well documented in humans (Hogarth et al., 2007; Bray et al., 2008; Talmi et al., 2008; Hogarth and Chase, 2011; Huys et al., 2011; Nadler et al., 2011; Hogarth, 2012) and both forms of transfer (specific and general) could play a role in instrumental approach behavior. For example, the sight of a beer brand logo may remind one of beer drinking which may activate approach behavior that is previously been instrumental in obtaining beer, via S–O–R associations. General PIT, on the other hand, can only further strengthen a pre-existing bias. For example, if there is already an approach bias toward alcohol, then any reward-associated cue (such as a cigarette logo for smokers) may further increase that bias by boosting the dominant approach response. Intriguingly, in animals, PIT effects have been shown to be insensitive to outcome devaluation (Rescorla, 1994; Holland, 2004) suggesting that these could play an important role in addiction relapse. Two recent studies in smokers provided evidence that Pavlovian cues predicting smoking outcomes increase the likelihood of responding for cigarettes and that furthermore, this can occur regardless of the current incentive value of the smoking outcome (Hogarth and Chase, 2011; Hogarth, 2012). In these studies, Pavlovian cues increased responding for cigarettes even after participants had read health warnings about cigarettes (Hogarth and Chase, 2011) or been treated with nicotine replacement therapy (Hogarth, 2012) and, crucially, had decreased responding in the absence of the cues. It seems paradoxical that this behavior, controlled by the anticipation of the outcome it produces, is not modulated by the current incentive value of that outcome, but this effect has been convincingly demonstrated in animals and humans (Rescorla, 1994; Holland, 2004; Hogarth and Chase, 2011; Hogarth, 2012; but see Allman et al., 2010). It appears, therefore, that in outcome-specific Pavlovian-instrumental interactions, the representation of the outcome contains sensory, but not motivationally relevant information (see Delamater and Oakeshott, 2007). The result is that the perceptual characteristics of the outcome prompt the associated response, regardless of the current incentive value of that outcome. Future research should elucidate the exact mechanism that mediates outcome-specific PIT, but on the basis of these outcome-reevaluation studies, we will make a distinction in the remainder of this manuscript between goal-directed action and outcome-specific PIT, with the latter also being mediated by anticipation of the outcome but occurring independently of the incentive goal status of the outcome.

## Theories of Addiction

The motivation driving destructive drug-seeking behavior is a key component of all major theories of addiction and most provide some explanation for why environmental cues can trigger relapse, even after long periods of sobriety. Some theories make clear predictions about the mechanisms that could facilitate an approach bias toward drug cues and whether such a bias is goal-directed, stimulus-bound habits, or due to PIT anticipatory processes. Relevant to the discussion at hand are incentive sensitization, theories based on the role of expectancy, various dual-process models (including habit theories of addiction), and negative reinforcement models.

### Incentive sensitization

The incentive sensitization model proposes that repeated drug use causes neuroadaptations in mesolimbic dopaminergic systems controlling the incentive values assigned to drug stimuli (Robinson and Berridge, 1993; Berridge, 2007). Over time a pathological incentive value becomes attributed to drug cues and contexts prompting compulsive drug-taking. This incentive sensitization increases even whilst levels of subjective pleasure decrease over the course of addiction (defined as increased “wanting” even in the absence of “liking” a drug). Thus whilst drugs can become disliked and an individual may have explicit motivations to avoid them, cues remain extremely salient and continue to elicit craving and motivate approach behavior (Robinson and Berridge, 1993, 2000, 2001).

### Expectancy theories

Some models propose that expectancies of drug outcomes play a crucial role in motivating drug-seeking behavior. Goldman and colleagues argue that drug use is a goal-directed choice, based on the expectation of the hedonic effect of the drug outcome (Goldman et al., 1987; Goldman, 2002). Following a meta-analysis of conditioning studies using tobacco rewards, Hogarth and Duka (2006) found evidence for the role of expectancies in drug-seeking behaviors. Recently, however, this view was extended, given demonstrations that cue-elicited anticipation of a cigarette reward prompted responding for that reward, even when incentive value was low. The authors suggested therefore that parallel goal-directed expectancies and PIT anticipatory processes jointly determine action control (Hogarth and Chase, 2011; Hogarth, 2012).

### Dual-process models

There are various dual-process models of addiction. Similarly to Tiffany’s habit theory of addiction (Tiffany, 1990; Tiffany and Conklin, 2000), associative theories make a distinction between goal-directed and stimulus-bound behaviors (Everitt et al., 2001; Everitt and Robbins, 2005; de Wit and Dickinson, 2009; Hogarth and Chase, 2011; Hogarth, 2012). Another group of dual-process models describe an automatic, appetitive system opposed by an executive control system (Strack and Deutsch, 2004; Wiers et al., 2007; Hofmann et al., 2008, 2009; Stacy and Wiers, 2010). These will be discussed in turn.

#### Habitual versus goal-directed control in dual-process models

Tiffany’s habit theory of addiction (Tiffany, 1990; Tiffany and Conklin, 2000) proposes that over time, drug-taking “rituals” become automatic behavioral schema, prompted by the environment. Whilst there are many unique features within Tiffany’s model, this transition from goal-directed to habitual behavior is also captured by associative dual-process models of addiction (Everitt et al., 2001; Everitt and Robbins, 2005). According to this view, the reinforcing effects of drugs lead to strong S–R associations between contextual stimuli and drug-seeking behaviors. Over time, approach behavior toward drugs becomes a habitual response, triggered by environmental cues. This behavioral transition appears to be paralleled by impaired functioning of cortico-striatal networks supporting goal-directed behavior (Porrino et al., 2004; Everitt and Robbins, 2005). In a slightly different vein, Hogarth and Chase (2011) argue that goal-directed and PIT anticipatory processes operate in an additive manner, jointly determining behavior.

#### Implicit versus explicit control in dual-process models

Another set of dual-process models are centered upon the notion that appetitive behavior can be automatically triggered by a variety of cues and that this behavior needs to be regulated by executive control processes, maintaining goal focus, and motivation to resist use and abuse of drugs (Strack and Deutsch, 2004; Wiers et al., 2007; Hofmann et al., 2008, 2009; Stacy and Wiers, 2010). Individual differences in impulsivity and cognitive control modulate the effectiveness of this regulation (Dawe et al., 2004; Hofmann et al., 2009; Peeters et al., 2012). The impulsive and executive dual processes can be mapped fairly well onto the stimulus-bound and goal-directed distinction of associative models, although it should be noted that the reflective control system is often argued to be propositional in nature, not associative (Strack and Deutsch, 2004).

#### Negative reinforcement theories

Alleviation of a negative affective state – either withdrawal symptoms or more generally, depression or stress – is a commonly cited cause of relapse (Carey and Correia, 1997; Shiffman and Waters, 2004; Kuntsche et al., 2005). Negative reinforcement theories highlight the role of internal cues (negative affective states) in prompting drug use (Koob and Le Moal, 1997; Baker et al., 2004; Eissenberg, 2004; Ahmed and Koob, 2005). Koob and colleagues propose that addiction is the result of dysfunction in not only the reward system but also the anti-reward system, driving aversive states (Ahmed and Koob, 2005; Koob and Le Moal, 2005). It is beyond the scope of the present paper to provide a detailed overview of negative reinforcement theories, but for the purposes of the current discussion we would like to note that similar motivational mechanisms may underlie drug-seeking based on the rewarding properties of drugs versus avoidance of aversive states (Baker et al., 2004; Eissenberg, 2004). Avoidance behavior in the context of drug use could be either a goal-directed strategy based on expectancies of the alleviating outcome, or a stimulus-response habit reinforced by alleviation of negative states, or the result of Pavlovian-instrumental interactions.

### Interim conclusions

Assessing the characteristics of the motivational mechanisms underlying addictive approach behavior, should provide evidence in favor of different models of addiction. These models overlap with respect to several common predictions. They all provide an explanation for why relapse can be triggered by environmental cues, whether this is due to S–R associations, incentive sensitization, or triggering of goal-directed expectancies. There are nonetheless a number of subtle distinctions. Many implicit/explicit dual-process models propose that the approach bias represents an automatic, positive evaluation of drug cues, which is argued to be distinct from explicit processes. Goal-directed expectancy theories on the other hand would argue that the bias arises due to positive expectancy of the drug outcome. Some unique predictions derive from these models, which can be empirically tested. For example, associative dual-process models predict that approach behavior will eventually be resistant to outcome devaluation as behavior transitions to habitual control. This is in stark contrast to goal-directed expectancy theories, which predict that decreases in outcome value will continue to reduce responding for drug outcomes.

## Empirical Data

A longstanding idea in psychological science is that a considerable amount of behavior is driven by rapid, evolutionary relevant, affective evaluations of stimuli. These affective evaluations classify all stimuli as either “negative” or “positive,” facilitating in the latter case, approach behavior (Bindra, 1974; Dickinson and Dearing, 1979; Chen and Bargh, 1999; Fazio, 2001; Krieglmeyer et al., 2010).

Motivations and affective attitudes are commonly assessed via questionnaires. Unfortunately, however, conclusions from such explicit measures can be difficult as participants may lack insight into the driving forces behind their actions and choices. In the case of addiction, invalid self-reports may result from self-presentational strategies or self-deception in an attempt to maintain a positive self-image. Moreover, introspection has been argued to not be a reliable and objective method of assessing motivational states (Berridge et al., 2009; Schooler and Mauss, 2009; Wood and Neal, 2009; Neal et al., 2012). As behavior becomes more habitual over time, there may not be a corresponding shift in subjective awareness of this fact. In the case of drug relapse, *post hoc* evaluation of one’s behavior may lead an individual to conclude that their behavior was motivated by a craving for the drug as opposed to being prompted by the external environment.

To overcome these difficulties and problems, a number of indirect, speeded reaction-time tasks have been developed to assess the valence and strength of affective evaluative associations (and the resulting approach behavior) without the need for explicit reflection on the part of the subject (Fazio, 2001; De Houwer, 2006; De Houwer et al., 2009). We focus here on measures of action tendencies, although it should be noted that varieties of the Implicit Association Task have also been used to assess approach and avoidance associations of a target category such as alcohol (Palfai and Ostafin, 2003; Ostafin and Palfai, 2006).

### Measurements of approach bias

In order to directly assess approach tendencies, a number of tasks have been developed that measure speed of approach toward (generally pictorial) stimuli. Approach bias is generally measured as the difference in reaction time on trials where participants make an approach movement (such as pulling a joystick) versus an avoid movement (pushing a joystick) to the pictorial stimuli on a computer screen. A number of tasks have been developed, and whilst they all measure approach bias, they are confusingly and interchangeably labeled as either stimulus-response compatibility (SRC) tasks (utilizing either a manikin or a joystick), approach avoidance tasks (AATs), or affective Simon tasks. To avoid confusion, we will use the explicit paradigm labels when describing these tasks in later sections.

### The manikin task

The manikin task provides an indirect measure of approach and avoidance behaviors. Approach tendencies are assessed by calculating the difference in reaction times across two blocks of experimental trials. In the first block the participant moves a computerized manikin toward one category of stimuli (e.g., alcohol) and away from other stimuli (e.g., soft drinks). In a subsequent block this assignment reverses. Using this task, participants have been seen to approach positive words faster than they are avoided, with the reverse effect for negative words (De Houwer et al., 2001). In addition, the manikin task has been used to assess approach behaviors in studies focusing on eating disorders (Woud et al., 2011), obesity (Havermans et al., 2011), Pavlovian conditioning of neutral stimuli (Thewissen et al., 2007; van Gucht et al., 2008), as well as approach tendencies toward alcohol (Field et al., 2005b, 2008; Schoenmakers et al., 2008; van Hemel-Ruiter et al., 2011; Barkby et al., 2012), cigarettes (Mogg et al., 2003, 2005; Bradley et al., 2004; Field et al., 2005a), and cannabis (Field et al., 2006; Cousijn et al., 2012).

### The joystick task

The joystick task can be used to measure differences in reaction times when the participant pushes a joystick away from his/her body in response to stimuli as opposed to pulling the joystick toward his/her body on a subsequent block. This task has been used to study phobias and anxiety (see Roefs et al., 2011), lifestyle and fitness goals (Fishbach and Shah, 2006), and food deprivation manipulations (Seibt et al., 2007).

Whilst it was originally suggested that approach and avoidance movements are represented as stored motor patterns, triggered by automatic, affective stimuli evaluations, it has became increasingly clear that motor actions *per se* do not represent either approach or avoidance. The same motor response (such as arm flexion) may represent approach in one situation (moving something toward oneself) but avoidance in another situation (quickly moving hand away from a stimulus to be avoided; Chen and Bargh, 1999). Indeed many studies have now shown that it is an individual’s interpretation of the result of the behavior that is important (i.e., is the stimulus moved closer or further away) and as such, neutral body movements can be interpreted as approach and avoidance actions depending on the outcome (Lavender and Hommel, 2007; Seibt et al., 2008; van Dantzig et al., 2008; Krieglmeyer et al., 2010). The zooming joystick task (ZJT) is thus a disambiguated version of the joystick task, designed to avoid misinterpretation and recategorization of pushing and pulling movements. The introduction of a zooming feature ensures that participants experience the illusion of stimuli moving away from them and coming toward them when they push or pull the joystick (this is achieved by reducing or enlarging the size of the picture). This zooming feature reduces the possibility of participants interpreting pulling movements as avoid rather than approach. Using the zooming version of the task (negative) approach tendencies to spiders have been assessed in spider phobia (Rinck and Becker, 2007).

By asking participants to respond to an irrelevant task feature such as orientation or location of the picture on screen instead of the content of the picture, the task may be rendered more implicit (De Houwer, 2003). Using the ZJT with *irrelevant feature instructions*, approach bias has been examined in heavy drinkers (Wiers et al., 2009, 2010), alcoholic patients (Wiers et al., 2011), at-risk adolescents (Peeters et al., 2012), and heavy cannabis users (Cousijn et al., 2011).

### Reliability and validity

Studies correlating approach bias measures to real-life behavior, as opposed to self-report measurements, are limited in number (due in part to the complexity of such designs). Rinck and Becker (2007) found that an approach bias on the ZJT predicted actual approach behaviors to live spiders, over and above that which was predicted with spider-phobia questionnaires. More importantly for the present discussion, approach bias on an irrelevant feature version of the ZJT was correlated with the amount of alcohol drunk in what was described to participants as an unrelated consumer “taste test” following the task (Wiers et al., 2010).

The split-half reliability of the manikin and joystick tasks is variable but generally good when using task-relevant feature instructions (Rinck and Becker, 2007; Krieglmeyer and Deutsch, 2010; Field et al., 2011). The advantage of instructing participants to respond on the basis of a task irrelevant feature is that it makes the task less susceptible to explicit control on the part of the participant, the practical drawback is that compatibility effects tend to be smaller (Krieglmeyer and Deutsch, 2010; Field et al., 2011). This reduced effect may be due to the fact that attention is not drawn to the affective properties of the stimuli in the irrelevant feature version. Several studies have, nonetheless, demonstrated robust approach (or avoidance) biases using irrelevant feature instructions (De Houwer et al., 2001; Rinck and Becker, 2007; Seibt et al., 2007; Wiers et al., 2009, 2010; Veenstra and de Jong, 2010; Cousijn et al., 2011). The reliability of the irrelevant feature instruction version was found to be poor in one study (Krieglmeyer and Deutsch, 2010) whilst another reported reasonably good reliability (Cousijn et al., 2011).

There are, evidentially, pros and cons to the various approach/avoidance task versions. Two studies, conducted with both the manikin and the standard joystick tasks, unexpectedly failed to find evidence for a correlation between the two approach bias scores (Krieglmeyer and Deutsch, 2010; van Hemel-Ruiter et al., 2011). The reasons for this are not immediately clear although there are major differences in how approach and avoidance are conceptualized within the tasks. Both the standard joystick task (without the zooming feature) and the manikin task are susceptible to recategorization – the manikin can be recategorized as someone other than the self and the approach/avoid movements in the standard joystick task are relatively ambiguous. Future studies should carefully select the task paradigm, depending on the research question, a point that we will return to in a later section “Outstanding Questions and Future Directions.”

### Approach biases in addiction

Using these aforementioned approach/avoidance paradigms, addiction researchers have provided substantial evidence for a relationship between drug-approach bias and drug use. That is, although the approach bias is measured experimentally in a lab, with superficial key press or lever movements, the behavioral tendency to be faster at approaching rather than avoiding drug stimuli, does seem to confer information about drug behavior more generally. Approach tendencies have been demonstrated in heavy (non-clinical) users of alcohol (Schoenmakers et al., 2008; Wiers et al., 2009, 2010), social drinkers (Field et al., 2005b), and cigarette smokers (Field et al., 2005a; Thewissen et al., 2007). Cigarette smokers have been seen to show a greater approach bias than non-smokers (Mogg et al., 2003; Bradley et al., 2004), as do cannabis users versus non-users (Field et al., 2006; Cousijn et al., 2011). Hazardous (non-clinical) drinkers were seen to have a stronger approach bias compared to light drinkers (Field et al., 2008). These results suggest a reliable relationship between drug use and drug-approach bias, particularly when examining healthy participants with moderate levels of dependence.

It should be noted that patterns of results can differ depending on the populations studied. Whilst lighter drinkers showed a weaker approach bias (Field et al., 2008), this pattern was reversed in one study investigating light versus heavy cigarette smokers (Mogg et al., 2005). In addition, in contrast to the aforementioned alcohol studies (with students), three studies involving patients receiving treatment for alcoholism did not find stronger approach tendencies for alcohol pictures compared to soft drink pictures (Wiers et al., 2011; Barkby et al., 2012; Spruyt et al., in press). These studies are small in number, but differing patterns of results in different populations at different stages of addiction can likely tell us something about the role of explicit motivations in approach bias measurements. We will discuss this in further detail, in the later section “Outstanding Questions and Future Directions.”

## Approach Tendencies in Addiction and the Underlying Motivations

The question remains as to which of the cognitive and motivational mechanisms outlined earlier in the “Theoretical Background” section contribute to the approach bias as measured in the lab. Evidence for the contribution of a Pavlovian component to experimental measures of the approach bias comes from studies that have shown that CS-reward learning quickly engenders an approach bias toward these novel CSs. Using different variations of AAT, approach bias has been conditioned toward novel Pavlovian stimuli predicting cigarette outcomes (Thewissen et al., 2007) and chocolate outcomes (van Gucht et al., 2008). A direct association between these stimuli and an instrumental approach response cannot mediate the approach bias to novel CSs, indicating therefore that Pavlovian mechanisms do play a role in the approach bias.

There is evidence, however, suggesting that the approach bias cannot be completely reduced to a purely Pavlovian conditioned response. As discussed previously, Hershberger (1986) showed that under conditions where chicks needed to make a withdrawal response in order to make a food bowl move toward them, they were unable to suppress the urge to approach the food bowl. This behavioral inflexibility provides evidence that the approach behavior of the chicks was predominantly controlled by a Pavlovian mechanism. In contrast to chicks, however, humans are perfectly well able to adapt their approach behavior. To our knowledge, the human equivalent of Hershberger’s experiment has not been conducted yet, but a recent study did employ a similar design. In a manikin task, participants were required to make an initial brief avoid movement in order to approach positive words and an initial brief approach movement to avoid negative words. Krieglmeyer et al. (2011) showed that even when the initial movement is avoidance, participants will still react faster if the final outcome is that the manikin approaches positive words. The reverse was true for avoiding negative words such that even if the initial movement was to approach a negative word, participants reacted faster if the final outcome was avoidance. This study suggests that the approach bias is more complex than being a mere Pavlovian approach response, as the final outcome (is the stimulus further away or closer) and not the initial direction of movement (toward or away from the stimuli), seems to influence reaction times.

Retraining studies with the joystick task provide further evidence for instrumental control over approach behavior (Kawakami et al., 2007, 2008; Wiers et al., 2010, 2011). For example, Wiers et al. (2010) presented the vast majority of a set of alcohol pictures in the push rather than pull format and found that retraining reduced the approach bias toward these pictures. The observation that participants can modify the bias following training (avoiding appetitive pictures) suggests that the bias is more than a conditioned response and shows a degree of flexibility that is in line with an instrumental account of the approach bias.

The results discussed above suggest that the approach response is not a purely Pavlovian response, although it is challenging to disentangle the relative contributions of Pavlovian and instrumental mechanisms using these paradigms. The question remains, nonetheless, as to whether the approach bias is flexibly modulated by changes in incentive value of the outcome or whether it is merely triggered by the drug stimuli. A number of studies have found that approach bias measurements increase in line with self-report craving scores, a result that is generally interpreted to suggest that approach behavior is sensitive to the current incentive value of the outcome (Field et al., 2005b, 2008; van Gucht et al., 2008). However, this correlative finding should be interpreted with caution as it does not necessarily imply a causal relationship between craving and the approach bias. Furthermore, other studies failed to find evidence for a relationship between craving and the approach bias (Mogg et al., 2003; Thewissen et al., 2007; Wiers et al., 2010; Cousijn et al., 2011). However, none of those studies really address whether behavior is *immediately* sensitive to a change in the incentive outcome value. So far, such outcome-reevaluation designs have yielded mixed results. Two studies, using the manikin task, manipulated craving by giving participants a placebo drink in one session and a dose of alcohol in another. Approach bias scores to alcohol pictures (Schoenmakers et al., 2008) and smoking pictures (Field et al., 2005a) were then compared between the two sessions (alcohol or placebo). Both studies found that self-reported craving was higher in the alcohol session but there was no difference in the approach bias scores – a null effect that although difficult to interpret, is more in line with the habitual account. These results are in contrast to a study using the standard joystick task, that examined the effects of satiety on the approach bias (Seibt et al., 2007). Participant’s responses to images of food were measured either before or after lunch and satiety did appear to reduce the bias in the non-deprived group, suggesting that approach behavior was driven by the current desire for food. Unfortunately, however, this study failed to include a neutral control picture condition, and we can therefore not ascertain whether hunger increased the approach movement toward food pictures specifically, or approach behavior generally. Still, these results suggest that this line of research should be extended further to critically assess motivational modulation of approach.

As discussed previously, some dual-process theories suggest that the approach bias results from an interaction between associative learning processes and explicit cognitive control processes. Barkby et al. (2012) provided correlational evidence for the importance of cognitive control, by testing patients receiving treatment for alcohol addiction on the manikin task. Their critical finding was that approach bias scores on the manikin task correlated with individual differences in explicit approach/avoidance intentions. Further evidence, that behavioral intentions can influence approach behavior, comes from a study using a variant of the ZJT (Sharbanee et al., in press). Rather than calculating approach bias as a difference score between the push and pull reaction times, this study made a distinction between “pull alcohol picture” trials and “push alcohol picture” trials – the former trial type assumed to be congruent with an appetitive tendency and the latter incongruent. Only incongruent trials, therefore, should demand recruitment of executive control processes (to overcome the appetitive tendency and push the alcohol picture away). As expected, results showed that working memory scores modulated reaction times in problem drinkers attempting (unsuccessfully) to control their alcohol consumption, but this effect was only observed on incongruent “push alcohol” trials. This suggests that the approach bias arises due to a complex interaction between the strength of the approach tendency and the ability to inhibit this tendency when required.

We should point out, however, that many other studies suggest that approach tendencies are not always under intentional control. These studies report seemingly “automatic” approach biases that are not in line with instrumental withdrawal intentions: participants scoring higher on a restrained eating scale showed a greater approach bias toward food cues (Veenstra and de Jong, 2010); smokers showed an approach bias toward smoking cues that they reported as unpleasant (Bradley et al., 2008); and appetitive Pavlovian stimuli inhibited instrumental withdrawal in situations where the instrumental withdrawal behavior was rewarded with money (Huys et al., 2011). It appears, therefore, that explicit intentions can sometimes influence approach, but a complete account of the approach bias will also have to encapsulate the role of associative learning processes.

To summarize, the evidence surveyed suggests that both Pavlovian and instrumental mechanisms play a role in facilitating the approach bias, but it is not yet clear how these processes interact or sum to produce this behavioral tendency. Furthermore it remains to be seen whether the approach bias is flexibly modulated by outcome value or has the characteristics of a habitual response to drug cues. Recent research examining instrumental responding for cigarette outcomes has argued that goal-directed and PIT processes operate in parallel, summing in an additive manner (Hogarth and Chase, 2011; Hogarth, 2012). However, the role of PIT in the approach bias remains to be empirically addressed. Furthermore, next to associative mechanisms, behavioral intentions may also modulate the approach bias, with one study suggesting that the approach bias measures some combination of both appetitive and regulatory control processes (Sharbanee et al., in press).

## Outstanding Questions and Future Directions

Approach bias tasks offer a fast and simple manner of measuring approach tendencies to drug-related stimuli and appear to tell us something about drug use, given that a number of studies have correlated approach bias scores with actual drug use (Field et al., 2008; Wiers et al., 2010; Cousijn et al., 2011) and shown group differences between heavier versus lighter/non-users (Mogg et al., 2003; Bradley et al., 2004; Field et al., 2006; Cousijn et al., 2011). Whilst understanding the mechanisms that underlie the approach bias is an important theoretical question, it should be noted that these tasks are not ideally suited to dissociating the various motivation mechanisms introduced in the earlier “Theoretical Background” section. To isolate goal-directed approach from Pavlovian approach for example, requires a task where the instrumental actions are bidirectional (i.e., left and right). In such a task, the relationships between the stimulus and the outcomes are held constant whilst the relationships between the *direction* of action and the outcomes are manipulated. The relative contribution of Pavlovian processes is equal to both actions, and hence controlled for (Dickinson et al., 1996). In addition, it has been observed that outcome devaluation modulates both conditioned Pavlovian responses and goal-directed instrumental responses (Colwill and Rescorla, 1988), and as such we cannot differentiate between these two in an outcome-reevaluation study if the approach bias is the dependent measure. Nonetheless, whether the approach bias is flexibly modulated by outcome reevaluation or is directly triggered by the drug stimuli is an outstanding question. The studies that have employed outcome-reevaluation paradigms have yielded mixed results (Field et al., 2005a; Seibt et al., 2007; Schoenmakers et al., 2008) and this line of research within the context of addictive substances, should be continued.

Specifically, outcome-reevaluation studies conducted with individuals at different levels of dependency, could address the question of whether the approach bias becomes more habitual over the course of addiction. Given the observation that users receiving clinical treatment may not show a very strong approach bias (Wiers et al., 2011; Barkby et al., 2012; Spruyt et al., in press), this method could also be used to assess whether control over approach behavior is regained during (successful) treatment. The work of Hogarth and colleagues in the field of smoking addiction suggests that there are a number of ways to reevaluate addictive substances, namely health warnings, temporary satiety through consumption, and treatments aimed at alleviating withdrawal symptoms (Hogarth and Chase, 2011; Hogarth, 2012). Another way may be to pair the consumption of an appetitive substance with an aversive flavor (Howard, 2001; van Gucht et al., 2010). In order to conduct an outcome-reevaluation test, an approach bias measurement would first be taken with neutral and category of interest (e.g., smoking) pictures. Then the smoking outcome would be devalued (e.g., through satiety) and the approach bias measurement would be repeated. If the approach bias measurement is not reduced following outcome devaluation, this would suggest that the approach bias is a stimulus-bound response to drug stimuli. Different versions of the approach bias tasks, as reviewed earlier, may be better suited to reevaluation studies given that ideally a repeated measures design is employed and that the second measurement, following outcome devaluation, should be conducted in extinction (without presentation of the outcome). The standard joystick or manikin tasks therefore, with irrelevant feature version, would be preferable in such a paradigm – as participant awareness of the study aims should be reduced as much as possible.

Pavlovian-instrumental interactions are thought to play an important role in addictive behavior (Hogarth and Chase, 2011; Hogarth, 2012). However, whether PIT processes *can* confer a specific or general motivating effect on approach/avoidance movements on these tasks has not been assessed. This could be investigated using, for instance, the manikin task. In an initial instrumental (O–R) learning phase, participants would make an approach movement to earn one specific outcome and avoidance to earn another (e.g., approach is rewarded with beer; avoidance rewarded with wine). This would be followed by Pavlovian (S–O) training where participants would learn the predictive relationship between neutral stimuli and these same outcomes. During the subsequent transfer test, occasional stimulus presentations would be expected to facilitate/speed up the response associated with a common outcome. For example, a stimulus for beer would be expected to facilitate approach, while a stimulus for wine should facilitate avoidance. General PIT, on the other hand, could be assessed by comparing the influence of a stimulus associated with a third outcome (e.g., whiskey) versus one associated with no alcoholic drink.

fMRI research of the approach bias is scarce, but given the wealth of knowledge that exists concerning the neural correlates of the various motivational mechanisms highlighted in this review (see Balleine and O’Doherty, 2009 for a detailed overview), this could be a very fruitful avenue for investigating many of the questions raised thus far. Approach bias to cannabis using a manikin task was recently investigated in an fMRI study with heavy cannabis users (Cousijn et al., 2012). Results suggested that ventromedial prefrontal cortex (vmPFC) was recruited during congruent “approach cannabis” blocks as opposed to incongruent “avoid cannabis” blocks. The vmPFC/orbitofrontal cortex, along with the caudate, have been consistently implicated in goal-directed action (Valentin et al., 2007; de Wit et al., 2009, 2012b). Similar prefrontal regions are argued to encode Pavlovian outcome values (Gottfried et al., 2002, 2003). The results of Cousijn and colleagues suggest, therefore, that the approach bias is driven by mechanisms flexibly modulated by outcome value, as opposed to habits – the latter being mediated not by prefrontal regions, but instead networks involving the posterior putamen and premotor cortex (Tricomi et al., 2009; Ashby et al., 2010; de Wit et al., 2012b). We should point out however that another fMRI study reported contradictory findings – namely that vmPFC was recruited on incongruent and not congruent trials (Roelofs et al., 2009). Important methodological differences could account for these differential results – Roelofs and colleagues used a standard joystick task with affective, non-drug-related facial stimuli (happy/unhappy faces) and the effects disappeared when participants were instructed to approach/avoid on the basis of gender rather than facial expression (irrelevant feature version). Future studies should hopefully be able to resolve these findings.

Research examining the neural correlates of approach tendencies would be best suited to approach bias paradigms with high internal reliability – such as the relevant feature version of the manikin task. A question of interest is whether networks implicated in goal-directed control versus habitual control are recruited, and whether this is different between groups who are at different stages of addiction. In humans, differential regions of the amygdala are thought to mediate general and outcome-specific PIT (Prévost et al., 2012) and their engagement in approach bias tasks could also be examined. fMRI can also be used to assess the role of brain regions such as the anterior cingulate, known to be important in overcoming response conflict and cognitive control more generally (reviews: Botvinick et al., 2004; Ridderinkhof et al., 2004), during approach bias tasks. Although the fMRI studies mentioned earlier examined contrasts of either congruent > incongruent or incongruent > congruent – reporting the results of both contrasts would be beneficial to test hypotheses relating to the interaction between appetitive responses and explicit cognitive control.

Pharmacological manipulations could also be employed to investigate the effect of neurotransmitter depletion on approach tendencies, with dopamine being an obvious candidate. Females submitted to dopamine precursor depletion, for example, were seen to rely more on habitual S–R knowledge at the expense of goal-directed O–R knowledge in a task designed to assess the relative balance of these two systems (de Wit et al., 2012a). A GABA antagonist (Baclofen), also thought to have effects via mediation of dopaminergic systems, reduces craving and consumption in alcohol, and cigarette addiction (Franklin et al., 2009; Gorsane et al., 2012) yet the effect on the approach bias has not been studied. Studies of this type would help with attempts to understand what, exactly, the approach bias represents and how it relates to other measures such as craving.

Finally, the extent to which these manipulations selectively affect approach versus avoidance (rather than the composite approach bias score) is worth investigation. Some studies have started to tease apart the relative contributions of “congruent” approach responses to appetitive stimuli as opposed to “incongruent” avoidance movements away from appetitive stimuli, with interesting insights (Roelofs et al., 2009; Barkby et al., 2012; Cousijn et al., 2012; Sharbanee et al., in press). By looking at these processes separately we can gain a better understanding of what the approach bias actually measures and what role various cognitive and motivational mechanisms play in producing this effect.

## Conclusion

Human behavior can be paradoxical, in that actions are initiated that are seemingly incongruent with an individual’s explicit motivations. This is most commonly observed in addiction, where maladaptive behavior (i.e., drug seeking) appears to be compulsive. Different theoretical approaches attempt to explain this behavior in different ways, with some suggesting that positive expectancies drive such behavior and others arguing that environmental stimuli can trigger behaviors incongruent with current goals and behavioral intentions. A number of studies have observed correlations between problematic drug use and approach bias scores (Field et al., 2008; Cousijn et al., 2011) suggesting that approach bias measurements can index everyday behaviors. Understanding the cognitive and motivational mechanisms that drive such an approach bias may therefore provide insight into both adaptive and maladaptive action control.

Determining the mechanisms underlying approach may have clinical implications. Cognitive therapy may be useful if expectancies and cognitive control are important determinants of approach. On the other hand, alternative approaches such as exposure response prevention therapy or counter-conditioning, that target the behavior directly, may be more appropriate if approach is stimulus-bound (e.g., van Gucht et al., 2010). Retraining the approach bias using the ZJT has been shown to be effective in reducing approach behavior toward alcohol cues (Wiers et al., 2010), and in a clinical population such retraining of the approach bias leads to a significantly smaller relapse rate at one-year follow-up compared to individuals receiving sham training (Wiers et al., 2011). A better understanding of the motivational mechanisms that underlie the approach bias, will also provide a better understanding of what exactly is being trained in this novel paradigm and how it can be better improved as a viable treatment.

Integration of the literature on approach bias, motivation, and associative learning provides a clear framework with which to identify and disentangle the relative contributions of various cognitive and motivational mechanisms underlying such maladaptive behavior. Whilst the literature surveyed suggests that both Pavlovian and instrumental mechanisms contribute to the approach bias, it remains to be elucidated exactly how they interact and sum to produce approach behavior. Hopefully further research assessing these questions will be forthcoming, within the limits that are inherent to such a task paradigm. Understanding the mechanisms that underlie an approach bias will provide a better understanding of the complex interplay of automatic processes, outcome expectancies, and behavioral intentions underlying human action control. This is not only theoretically important but ultimately has implications for clinical treatment.

## Conflict of Interest Statement

The authors declare that the research was conducted in the absence of any commercial or financial relationships that could be construed as a potential conflict of interest.
